# Molecular characterization and antimicrobial resistance of *Enterococcus faecalis* isolated from seafood samples

**DOI:** 10.1002/vms3.761

**Published:** 2022-02-13

**Authors:** Neda Noroozi, Hassan Momtaz, Elahe Tajbakhsh

**Affiliations:** ^1^ Department of Microbiology Shahrekord Branch Islamic Azad University Shahrekord Iran; ^2^ Department of Microbiology Shahrekord Branch Islamic Azad University Shahrekord Iran

**Keywords:** antimicrobial resistance, Enterococcus faecalis, RAPD‐PCR typing, seafood, virulence factors

## Abstract

**Background:**

*Enterococcus faecalis* is considered an opportunistic foodborne pathogen. The present study aimed to assess the prevalence, antimicrobial resistance, virulence characters, and molecular typing of *E. faecalis* strains isolated from seafood samples.

**Methods:**

Two hundred and seventy‐six seafood samples were collected. *E. faecalis* was isolated from samples using bacterial culture. Furthermore, the disk diffusion assessed their antimicrobial resistance. Also, the distribution of virulence factors was determined using polymerase chain reaction (PCR) assay. Random amplified polymorphic DNA (RAPD) method was used for their molecular typing.

**Results:**

Fifty‐six of 276 (20.2%) seafood samples were contaminated with *E. faecalis*. Fish harboured the highest contamination rate (30.0%). Isolates harboured the highest resistance rate towards oxacillin (100%), tetracycline (100%), erythromycin (100%), cefoxitin (89.2%), cefazolin (87.5%), trimethoprim‐sulfamethoxazole (85.7%), rifampin (69.6%), clindamycin (69.6%), and gentamicin (64.2%) antimicrobials. *Efa* (100%), *ebpA* (89.2%), *ebpB* (58.9%), *ebpC* (53.5%), and *esp* (51.7%) were the most commonly detected virulence factors among *E. faecalis* isolates. RAPD–PCR analysis showed 11 different molecular clusters considering the closeness of more than 80%.

**Conclusion:**

Seafood samples were considered reservoirs of virulence and resistant *E. faecalis* strains. Different molecular clusters of isolates may reflect their diverse sources of contamination.

## Graphical abstract

*Enterococcus faecalis* bacteria were isolated from fish, shrimp, and lobster samples using the microbial culture. Antimicrobial resistance pattern of *E. faecalis* isolates was evaluated using the disk diffusion method. The molecular typing of *E. faecalis* isolates was assessed by Random amplified polymorphic DNA (RAPD)‐PCR.

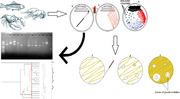

## 1 INTRODUCTION

*Enterococcus faecalis* bacteria are important microorganisms of humans’ and farm animals’ gastrointestinal tracts (Vu & Carvalho, 2011). *E. faecalis* strains can survive in hot, salty, or acidic environments (Byappanahalli et al., 2012). Additionally, they can easily be adapted to the gastrointestinal tract of their hosts and mainly found in the soil, water, and the environment (Daniel et al., 2017). *E. faecalis* infections are primarily spread from person to person through poor hygiene. For this reason, these bacteria are found in faces. The bacteria can get into foods through inadequate hygiene and food manipulation. Foodstuffs, particularly ready‐to‐eat food samples and those with animal origins, maybe the sources of bacterial transmission (Ali et al., 2017; Al‐Zubidi et al., 2019; Chapman et al., 2020; Fiore et al., 2019; Hammerum 2012; Hanchi et al., 2018).

*E. faecalis* strains cause serious infections, including gastrointestinal and urinary tract infections, meningitis, bacteraemia, and periodontitis (Abat et al., 2016; Ma et al., 2021; Prajsnar et al., 2013; Said et al., 2021). The severity and pathogenicity of diseases caused by these bacteria are higher in the presence of well‐defined virulence factors and toxins (Goh et al., 2017; Wu et al., 2020). Clinical investigations showed that enterococcal surface protein (*esp*), Fsr regulator locus responsible for bacterial quorum sensing (encoded by *fsrA*, *fsrB*, and *fsrC* genes), structural pilin genes (*ebpA, ebpB*, and *ebpC*), cytolysin (*cylL* and *cylS*), and endocarditis‐specific antigen (*efa*) are the most important virulence factors of the bacterium responsible for adherence, colonization, evasion, enzymes extracellular production, biofilm development and pathogenicity, and severity of subsequent infections (Goh et al., 2017; Bin‐Asif & Abid Ali, 2019). Destructiveness variables of *Enterococcus* spp. may contribute to competition with other microbes, colonization of the have, resistance against defence instruments of the have, and generation of obsessive changes specifically through the generation of poisons or by implication through acceptance of aggravation (Kayaoglu & Ørstavik, 2004). Infections caused by *E. faecalis* are mainly hard to treat by common antimicrobials (Shiadeh et al., 2019). Surveys showed the high resistance rate of *E. faecalis* clinical strains towards commonly used antimicrobials, particularly penicillins, tetracyclines, aminoglycosides, phenicols, cephalosporins, penicillins, and macrolides (Ahmed & Baptiste, 2018). Therefore, the assessment of antimicrobial resistance of *E. faecalis* strains can directly introduce the most suitable antimicrobial agents for further therapeutic options (Johnston & Jaykus, 2004; Perera et al., 2020).

According to the high pathogenicity of *E. faecalis* as an opportunist foodborne pathogen and the absence of epidemiological surveys in this field, the present research was performed to evaluate the prevalence, antimicrobial resistance, virulence factors distribution, and molecular typing of *E. faecalis* bacteria isolated from seafood samples.

## 2 MATERIALS AND METHODS

### 2.1 Sampling

Through the summer of 2020, a total of 276 seafood samples, including fish (*Scomberomorus commerson*) (*n* = 120), shrimp (*Penaeus indicus*) (*n* = 120), and lobster (*Panulirus homarus*) (*n* = 36), were collected from shopping centres of the Isfahan province, Iran. Seafood species identification was performed by a professor of aquatic research in the Islamic Azad University, Shahrekord Branch, Iran. All samples were caught from the Persian Gulf. For this purpose, the dorsal muscles of seafood samples were selected for sample collection. Samples (100 g) were transferred to the laboratory immediately at 4°C using separate sterile plastic bags.

### 2.2 *E. faecium* isolation and identification

Twenty‐five grams of each seafood sample was homogenized in 225 ml of sterile tryptone soy broth (TSB; Merck, Germany) using Stomacher Bagmixer 400 W (Interscience, Saint‐Nom, France) for 2 min. Cultures were incubated at 37°C for 24 h. A 5 ml aliquot of the enriched homogenate was transferred into 50 ml of bile esculin agar (Merck) and incubated at 37°C for 24 h. Two distinct colonies with black hallow characteristics on the bile esculin agar were purified on nutrient agar (Merck) and incubated at 37°C for 24 h. Isolated identification was performed using Gram staining, colony morphology, grow in hypersaline medium catalase test, and bile esculin reaction (Igbinosa & Beshiru, 2019). Finally, *E. faecalis* identification was performed using the polymerase chain reaction (PCR) (Klibi et al., 2015). *E. faecalis* (ATCC 19433) was used as a positive control.

### 2.3 Antimicrobial resistance

*E. faecalis* isolates (with 0.5 McFarland concentration) were aerobically cultured on the Mueller–Hinton agar (Merck) containing antimicrobial disks and further incubated at 37°C for 24 h. Antimicrobial resistance of *E. faecalis* isolates was assessed towards penicillin (10 μg/disk), oxacillin (5 μg/disk), gentamicin (10 μg/disk), erythromycin (15 μg/disk), tetracycline (30 μg/disk), levofloxacin (5 μg/disk), clindamycin (2 μg/disk), trimethoprim‐sulfamethoxazole (25 μg/disk), chloramphenicol (30 μg/disk), rifampin (5 μg/disk), nitrofurantoin (100 μg/disk), cefazolin (30 μg/disk), cefoxitin (30 μg/disk), clindamycin (2 μg/disk), norfloxacin (10 μg/disk), daptomycin (30 μg/disk), and linezolid (10 μg/disk) antibiotic agents (Padtanteb, Iran) (Dehkordi, Barati, et al., 2013; Dehkordi, Gandomi, et al., 2017). The instructions of Clinical and Laboratory Standards Institute (CLSI) were used for interpretation (CLSI, 2015). *E. faecalis* (ATCC 19433) was used as a positive control.

### 2.4 Virulence factors detection

*E. faecalis* isolates were sub‐cultured on TSB media (Merck) and further incubated for 48 h at 37°C. According to the manufacturer's instructions, genomic DNA was extracted from bacterial colonies using the DNA extraction kit (Fermentas, Germany). Purity (A260/A280) and concentration of extracted DNA were then checked (NanoDrop, Thermo Scientific, Waltham, MA, USA) (Dehkordi et al., 2011a; Dehkordi, Saberian, et al., 2012; Dehkordi, Momtaz, et al., 2012). The DNA quality was assessed on a 2% agarose gel stained with ethidium bromide (0.5 μg/ml) (Thermo Fisher Scientific, St. Leon‐Rot, Germany) (Dehkordi, Haghighi, et al., 2013; Dehkordi, Yazdani, et al., 2014). Table 1 represents the list of primers used to amplify the *E. faecalis* virulence factors (Eaton & Gasson, 2001; Hashem et al., 2017). A programmable DNA thermo‐cycler (Eppendorf Mastercycler 5330; Eppendorf‐Nethel‐Hinz GmbH, Hamburg, Germany) was used in all PCR reactions. Ten microliters of PCR product were exposed to electrophoresis in a 2% agarose gel in 1X TBE buffer at 80 V for 30 min, stained with SYBR Green. The UVI doc gel documentation systems (Grade GB004; Jencons PLC, London, UK) were applied to analyze images (Ghorbani et al., 2016; Dehkordi et al., 2020).

**TABLE 1:** Polymerase chain reaction (PCR) primers for virulence factors detection in *E. faecalis* isolates

Target gene	Primer sequence (5′‐3′)	PCR product (bp)	References
*efa*	F: GACAGACCCTCACGAATA R: AGTTCATCATGCTGTAGTA	705	Eaton and Gasson (2001)
*esp*	F: TTGCTAATGCTAGTCCACGACC R: GCGTCAACACTTGCATTGCCGAA	933	
*ebpA*	F: CCATTTGCAGAAGCAAGAATG R: GAGTGAAAGTTCCTCCTCTAG	613	Hashem et al. (2017)
*ebpB*	F: CATTAGCAGAGGCATCGCAA R: CAAGTGGTGGTAAGTCATAGG	504	
*ebpC*	F: CTGCTACGAATATGGTGGTG R: GGTGTTTGATTGTTTGCTTC	487	
*cylL*	F: GATGGAGGGTAAGAATTATGG R: GCTTCACCTCACTAAGTTTTATAG	253	Semedo et al. (2003)
*cylS*	F: TGCTAAATAAGGAAAATCAAG R: CCTAAGCCTATGGTAAAACA	157	Hällgren et al. (2009)
*fsrA*	F: CGTTCCGTCTCTCATAGTTA R: GCAGGATTTGAGGTTGCTAA	474	Versalovic and Lupski (2002)
*fsrB*	F: TAATCTAGGCTTAGTTCCCAC R: CTAAATGGCTCTGTCGTCTAG	428	
*fsrC*	F: GTGTTTTTGATTTCGCCAGAGA R: TATAACAATCCCCAACCGTG	716	

### 2.5 Molecular typing

Random amplified polymorphic DNA (RAPD)‐PCR analysis was done using the primer M13(5′‐GAGGGTGGCGGTTCT‐3′) as described previously (Versalovic & Lupski, 2002). Grouping of the RAPD‐PCR patterns was performed using the UPGMA cluster analysis. The strains grouping coefficients of similarity of 80% for RAPD typing were applied.

### 2.6 Statistical analysis

Data obtained in this survey were analyzed using the SPSS 21.0 software (SPSS Inc., Chicago, IL, USA). For this purpose, data were analyzed by *χ* ^2^ test and Fisher's exact two‐tailed tests, and significant relationships and differences between data were determined. *p*‐Value < 0.05 was considered as a statistically significant level (Dehkordi et al., 2011b; Dehkordi, Parsaei, et al., 2012; Dehkordi, Haghighi Borujeni, et al., 2013; Dehkordi, Valizadeh, et al., 2014; Dehkordi, Khamesipour, et al., 2014; Dehkordi, Tirgir, et al., 2017).

## 3 RESULTS

### 3.1 *E. faecalis* prevalence

Table 2 shows the *E. faecalis* prevalence among seafood samples. Fifty‐six out of 276 (20.2%) seafood samples were contaminated with *E. faecalis*. Fish samples harboured the highest contamination rate (30.0%), while lobster samples harboured the lowest (5.5%). Statistically, a significant relation was obtained between seafood types and *E. faecalis* prevalence (*p* < 0.05).

**TABLE 2:** The prevalence rate of *E. faecalis* among seafood samples

Seafood samples	No. of collected samples	No. of samples positive for *E. faecalis* (%)
Fish	120	36 (30.0)
Shrimp	120	18 (15.0)
Lobster	36	2 (5.5)
Total	276	56 (20.2)

### 3.2 Antimicrobial resistance

**FIGURE 1:**
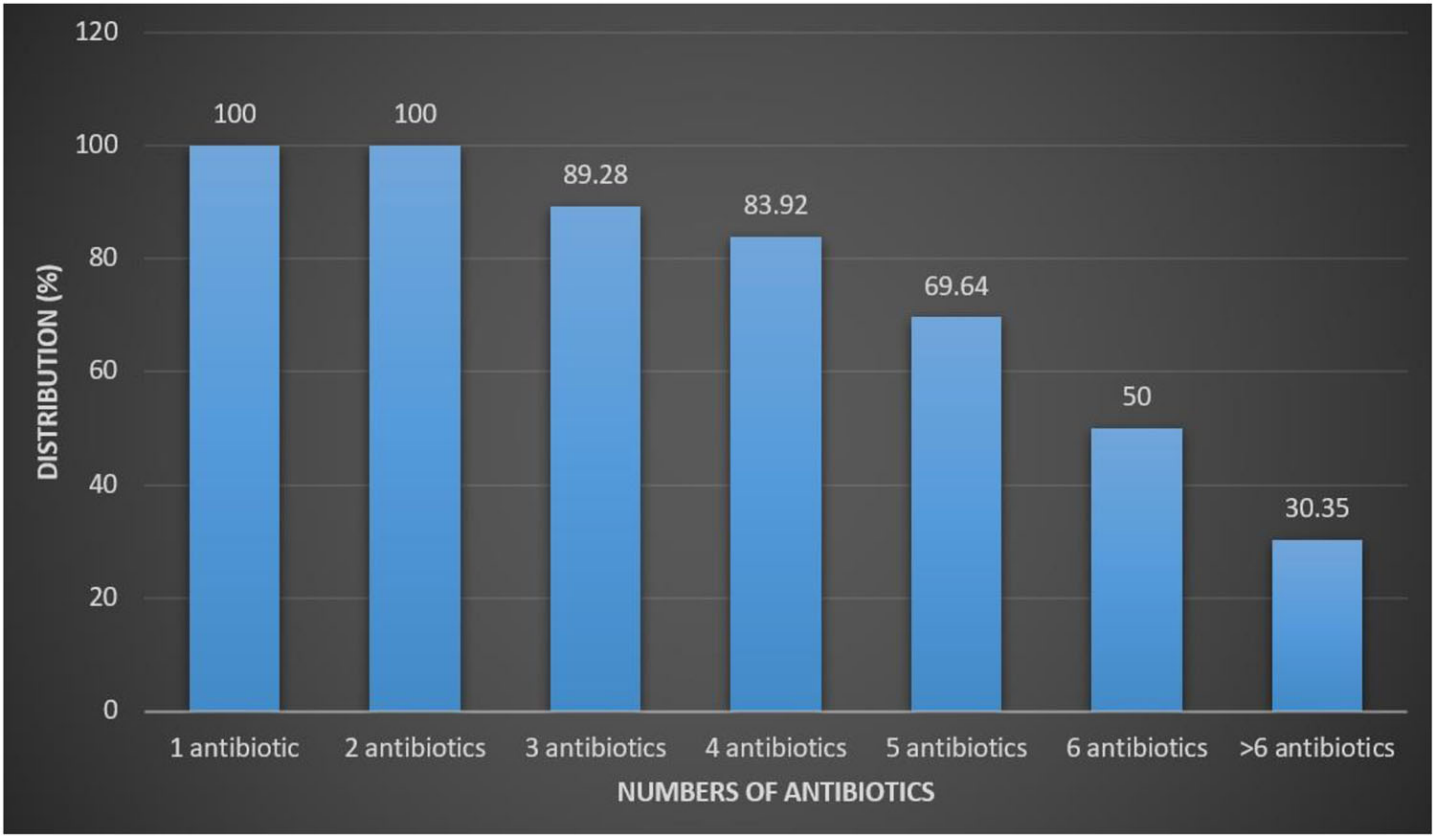
Distribution of multidrug‐resistant *E. faecalis* strains isolated from seafood samples

Table 3 shows the antimicrobial resistance pattern of *E. faecalis* bacteria isolated from seafood samples. *E. faecalist* isolates harboured the highest resistance rate towards oxacillin (100%), tetracycline (100%), erythromycin (100%), cefoxitin (89.2%), cefazolin (87.5%), trimethoprim‐sulfamethoxazole (85.7%), rifampin (69.6%), clindamycin (69.6%), and gentamicin (64.2%) antimicrobials. *E. faecalist* isolates did not show any resistance towards linezolid, nitrofurantoin, and chloramphenicol antimicrobials. The lowest resistance rate was obtained towards daptomycin (5.3%), vancomycin (10.7%), and norfloxacin (16.0%) antimicrobials. Statistically, a significant relation was obtained between seafood types and *E. faecalis* antibiotic resistance rates (*p* < 0.05). Figure 1 shows the distribution of multidrug‐resistant strains. As shown, all isolates had resistance to at least two different antibiotic agents. Findings showed that 30.35% of *E. faecalis* had resistance against more than six antibiotic agents.

**TABLE 3:** Antimicrobial resistance pattern of *E. faecalis* bacteria isolated from seafood samples

	No. of isolates harboured resistance against each antimicrobial agent (%)																	
Seafood samples (No. of positive)	G10	Cfz	Cfx	Ox	Tri‐Sul	Cln	Tet	Ert	Rif	Nor	Pen	Lev	Amp	Van	Dap	Lnz	Nit	C30
Fish (36)	36 (100)	33 (91.6)	35 (97.2)	36 (100)	32 (88.8)	30 (83.3)	36 (100)	36 (100)	33 (91.6)	7 (19.4)	29 (80.5)	8 (22.2)	9 (25.0)	2 (5.5)	3 (8.3)	–	–	–
Shrimp (18)	18 (100)	16 (88.8)	14 (77.7)	18 (100)	16 (88.8)	8 (50.0)	18 (100)	18 (100)	5 (31.2)	2 (12.5)	3 (18.7)	5 (31.2)	7 (43.7)	3 (18.7)	–	–	–	–
Lobster (2)	2 (100)	–	1 (50)	2 (100)	–	1 (50)	2 (100)	2 (100)	1 (50)	–	–	1 (50)	–	1 (50)	–	–	–	–
Total (56)	56 (64.2)	49 (87.5)	50 (89.2)	56 (100)	48 (85.7)	39 (69.6)	56 (100)	56 (100)	39 (69.6)	9 (16.0)	32 (57.1)	14 (25.0)	16 (28.5)	6 (10.7)	3 (5.3)	–	–	–

Abbreviations: Amp, ampicillin; Cfx, cefoxitin; Cfz, cefazolin; Cln, clindamycin; C30, Chloramphenicol; Dap, daptomycin; Ert, erythromycin; G10, gentamicin; Lev, levofloxacin; Lnz, linezolid; Ox, oxacillin; Nit, nitrofurantoin; Nor, norfloxacin; Pen, penicillin; Rif, rifampin; Tri‐Sul, trimethoprim‐sulfamethoxazole; Tet, tetracycline.

### 3.3 Virulence factors distribution

Table 4 shows the virulence factors distribution among *E. faecalis* bacteria isolated from seafood samples. *Efa* (100%), *ebpA* (89.2%), *ebpB* (58.9%), *ebpC* (53.5%), and *esp* (51.7%) were the most commonly detected virulence factors among *E. faecalis* isolates. *FsrC* (28.5%), *cylL* (33.9%), *fsrB* (35.7%), and *fsrA* (44.6%) had the lowest distributions compared to other virulence factors. Statistically, a significant relation was obtained between seafood types and *E. faecalis* virulence factors profile (*p* < 0.05).

**TABLE 4:** Virulence factors profiles of the *E. faecalis* bacteria isolated from seafood samples

Seafood samples (No. of positive)	No. of isolates harboured each virulence factor (%)									
	*efa*	*ebpA*	*ebpB*	*ebpC*	*esp*	*cylL*	*cylS*	*fsrA*	*fsrB*	*fsrC*
Fish (36)	36 (100)	32 (88.8)	28 (77.7)	22 (61.1)	21 (58.3)	12 (33.3)	14 (38.8)	18 (50.0)	16 (44.4)	10 (27.7)
Shrimp (18)	18 (100)	17 (94.4)	5 (27.7)	7 (38.8)	8 (44.4)	6 (33.3)	6 (33.3)	7 (38.8)	3 (16.6)	4 (22.2)
Lobster (2)	2 (100)	1 (50)	–	1 (50)	–	1 (50)	2 (100)	–	1 (50)	2 (100)
Total (56)	56 (100)	50 (89.2)	33 (58.9)	30 (53.5)	29 (51.7)	19 (33.9)	22 (39.2)	25 (44.6)	20 (35.7)	16 (28.5)

### 3.4 RAPD‐PCR typing

**FIGURE 2:**
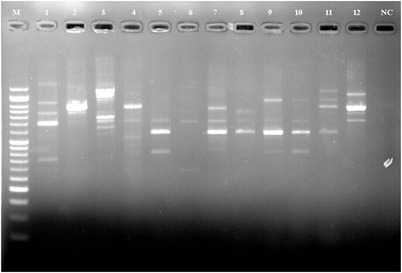
Polymerase chain reaction (PCR) gel electrophoresis of *E. faecalis* isolates in the RAPD analysis

**FIGURE 3:**
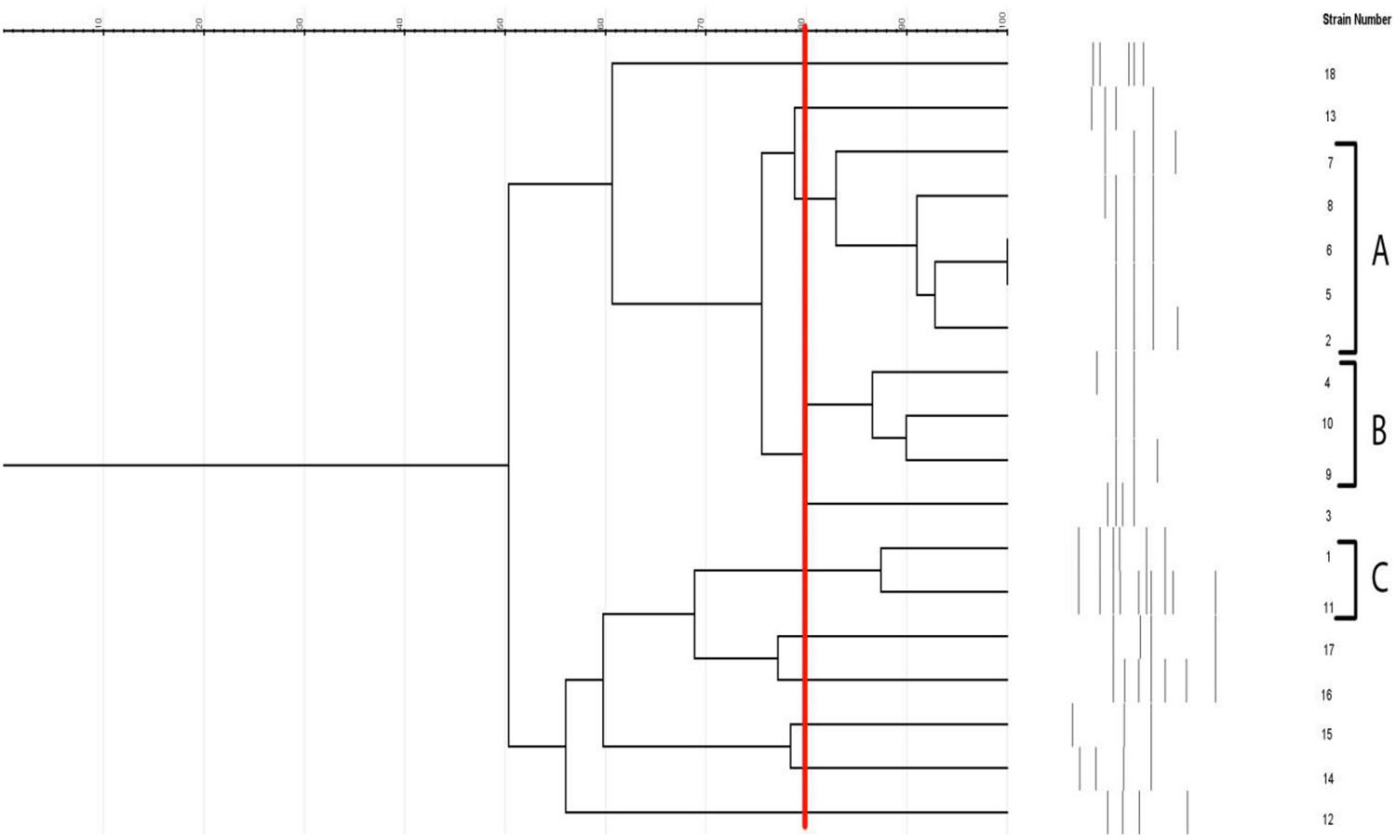
Random amplified polymorphic DNA–polymerase chain reaction (RAPD–PCR) molecular typing of *E. faecalis* isolates

Figure 2 shows the PCR gel electrophoresis of *E. faecalis* isolates in the RAPD analysis. The pattern of RAPD‐PCR‐based classification is shown in this figure. Figure 3 shows the RAPD‐PCR‐based typing of *E. faecalis* isolates. In the analysis of 18 isolates with RAPD marker, the isolates were placed in 11 profiles considering the closeness of more than 80%, among which isolates 3, 12–18 were placed in a separate profile. Profile A with five isolates 2, 5–8 is considered as the dominant clone. Isolates 5 and 6 in this category have 100% affinity.

## 4 DISCUSSION

The present study was performed to assess the prevalence, antimicrobial resistance, virulence factors characterization, and molecular typing of *E. faecalis* strains isolated from seafood samples. Findings showed that 20.2% of examined seafood samples were contaminated with *E. faecalis*. In comparison with our findings, Ellis‐Iversen et al. (2020) stated that *E. faecalis* was detected in 87.0% of pangasius fillets and prawns in Danish retail imported from Asia. *E. faecalis* prevalence in fish samples from Brazil was 44.3% (Araújo et al., 2021). Surveys conducted by Di Cesare et al. (2013), Do Vale Pereira et al. (2017), and Novais et al. (2018) reported that seawater and sediment samples were the main sources of seafood contamination with *E. faecalis* strains. However, cross‐contamination by human manipulation through fishing, transport, storage, and sale are introduced as risk factors for *E. faecalis* occurrence in seafood samples (Shikongo‐Nambabi et al., 2011). The prevalence rate of *E. faecalis* in seafood samples in Egypt (Ahmed et al., 2021), Switzerland (Boss et al., 2016), Lybia (Naas et al., 2017), and Nigeria (Igbinosa & Beshiru, 2019) was 7.0%, 59.0%, 70.2%, and 8.1%, respectively. Similar to our report, Pesavento et al. (2014) and Chajecka‐Wierzchowska et al. (2016) showed that *E. faecalis* was the most prevalent *Enterococci* among foodstuffs. This is the first report of isolation of *E. faecalis* in lobster samples, to the best of our knowledge. The higher prevalence of *E. faecalis* in fish samples may be attributed to the higher catch rate of fish than shrimp and lobster, resulting in lower hygienic conditions and the possibility of cross‐contamination between fish samples. Put together, caution is advised concerning the origin of isolated *E. faecalis*. The tested samples were purchased at retail, and the high prevalence of *E. faecalis* might be a sign of human and animal faecal contamination of the aquaculture environment or acquired during processing because these bacteria are not part of the normal bacterial flora of fish, shrimp, and lobster.

Most isolates in this study were resistant to common antimicrobial agents used in Iran, particularly oxacillin, tetracycline, erythromycin, cefoxitin, cefazolin, trimethoprim‐sulfamethoxazole, rifampin, clindamycin, and gentamicin. Irregular and unauthorized prescription of antimicrobial agents is the probable reason for the high resistance rate. As some isolates harbored a high resistance towards human‐based antimicrobial agents (those are basically used to treat human infectious diseases), it can be indirectly concluded that these isolates originated from infected staffs of seafood sales and processing centres (Ranjbar et al., 2019). The high resistance rate of *E. faecalis* strains isolated from food samples towards oxacillin, tetracycline, erythromycin, cefoxitin, cefazolin, trimethoprim‐sulfamethoxazole, rifampin, clindamycin, and gentamicin antimicrobial agents was reported from Switzerland (Boss et al., 2016), Turkey (Sanlibaba et al., 2018), Slovakia (Kročko et al., 2011), Africa (Olawale et al., 2015), Poland (Cybulska & Krzyśko‐ŁUpicka, 2020), and South Korea (Kim et al., 2021). Sergelidis et al. (2013) stated that *Enterococcus* isolates of fish and fish market samples harbored the high resistance rate towards cephalosporins, penicillins, and erythromycin antimicrobial agents. Karimian et al. (2018) and Samani et al. (2021) stated that *E. faecalis* isolates of meat samples harbored a high resistance towards streptomycin, cefotaxime, meropenem, erythromycin, and tetracycline (50%–70%) antimicrobials. Badul et al. (2021) displayed that *E. faecalis* resistance rate against ciprofloxacin, gentamicin, streptomycin, teicoplanin, quinupristin‐dalfopristin, nitrofurantoin, sulphamethoxazole‐trimethoprim, erythromycin, tetracycline, chloramphenicol, and levofloxacin antimicrobials was 9.3%, 15.1%, 69.8%, 0.0%, 3.1%, 77.8%, 71.6%, 79.6%, 25.3%, and 4.4%, respectively. *E. faecalis* strains isolated from food samples in Brazil (Riboldi et al., 2009) harbored the boost resistance rate towards ampicillin (11.1%), vancomycin (3.7%), erythromycin (11.1%), tetracycline (33.3%), ciprofloxacin (7.4%), norfloxacin (0.0%), nitrofurantoin (0.0%), chloramphenicol (7.4%), gentamicin (22.2%), and lincomycin (51.9%) antimicrobials. In Estonia (Aasmäe et al., 2019), *E. faecalis* resistance rate towards erythromycin, gentamicin, tetracycline, chloramphenicol, vancomycin, and linezolid antimicrobials was 10.3%–37.9%, 1.6%–5.7%, 20.5%–52.4%, 8.9%–13.3%, 0.63%–17.6%, and 0.0%–5.0%, respectively. Findings showed that all isolates were susceptible to linezolid and nitrofurantoin. This finding is probably due to the deficient administration of these two antibiotics, making bacterial isolates sensitive to them.

Several reports showed that the high distribution of some virulence factors, particularly *efa*, *ebpA*, *ebpB*, *ebpC*, and *esp* in the *E. faecalis* strains, guaranteed the high pathogenicity and clinical disease occurrence by these strains (Chen et al., 2018; Stępień‐Pyśniak et al., 2019). Savaşan et al. (2016) stated that *AS* and *gelE* virulence factors were detected in 26.9% and 11.5% of *E. faecalis* isolates of fish samples in Turkey. Araújo et al. (2021) stated that the distribution of *ace*, *agg*, *cylA*, *esp*, and *gelE* virulence factors among the *E. faecalis* isolates of fish farms was 88.5%, 22.8%, 8.5%, 0.0%, and 97.1%, respectively. Unlike the present study, the *esp* gene was not detected in the isolates of Brazilian research (Araújo et al., 2021). Ahmad et al. (2014) reported the distribution of *gel*E, *asa*, *esp*, and *cyl*A virulence factors in 100%, 63.4%, 21.9%, and 7.32% of *E. faecalis* isolates of aquatic environments in Malaysia. Aquatic environments present a large flow of water movement, essential for the renovation or filling of the ponds. Thus, it is possible to assume that virulence factors responsible for microbial adhesion (such as *ebp*) are useful to bacterial maintenance in this environment. Authors of the Nigerian survey (Igbinosa & Beshiru, 2019) reported that *gel*E (30.5%), serine protease (*sprE*) (32.2%), *cyl*L (10.2%), aggregation substance (*agg*) (62.7%), sex pheromones (*cpd* [61.0%, *cob* [98.3%], and *ccf* [94.9%]), cell wall adhesins (*efa*A) (77.9%), surface protein (*esp*) (98.3%), surface adhesion (*ace*) (79.7%), and hyaluronidase (*hyl*) (74.6%) were the most commonly detected virulence factors among the *E. faecalis* strains of seafood samples. Similar findings were reported by Han et al. (2011) and Barbosa et al. (2014). Jahan and Holley (2014) reported the high distribution of *esp, efa, gel*E, *ace*, and *agg* virulence determinants in *E. faecalis* recovered from meat products. Most isolates in this research harbored *esp* and *efa* virulence factors. Both factors are responsible for bacterial persistence and colonization in host cells. *Esp* gene was also detected in some isolates. This gene is responsible for the pathogenicity island, biofilm formation, and dynamics of antibiotic release (Leavis et al., 2004). *Esp* gene also had some effects in the occurrence of ampicillin, ciprofloxacin, and imipenem resistance (Billström et al., 2008) and vancomycin resistance (Ochoa et al., 2013) in *Enterococcus* strains. The role of *fsr* complexes in biofilm formation was also reported previously (Cybulska et al., 2020). In an Indian survey (Biswas et al., 2019), *cpd* and *efaAfs* were detected in all *E. faecalis* isolates of fish samples, and *gelE*, *agg*, and *esp* were detected in 17, 13 and 4 isolates, while *cylA* was not detected. Abou Zeid et al. (2019) and Adeniji et al. (2020) mentioned that *EF3314* (100%), *asa1* (87.5%), and *esp* (37.5%) virulence factors were detected in fish samples in Egypt.

Molecular typing of *E. faecalis* isolates showed 11 different profiles of molecular typing. This finding may show different origins of *E. faecalis* isolates of the present survey. The applied RAPD‐PCR method was also reported to be sensitive and practical for the molecular typing of clinical isolates of *E. faecalis* (Banerjee, 2013; Emaneini et al., 2016).

## 5 CONCLUSION

*E. faecalis* strains were detected in fish, shrimp, and lobster samples collected from the Persian Gulf. Isolates harbored both antibiotic resistance and virulence markers, which may show their high pathogenicity. In addition, isolates were classified into 11 different genetic clusters, showing their different sources of contamination. Considering the simultaneous presence of antibiotic resistance and virulence markers in some *E. faecalis* strains, the role of seafood samples as reservoirs of the bacteria and antibiotic resistance should be considered. Furthermore, rendering the latent relationship between virulence factors and antibiotic resistance in *E. faecalis* isolates, further studies should evaluate the role of genetic markers in the antimicrobial resistance properties of bacteria.

## CONFLICT OF INTEREST

The authors declare no conflict of interest.

## ETHICS STATEMENT

The research was extracted from the Ph.D thesis in the field of Microbiology and was ethically approved by the Council of Research of the Faculty of Basic Science, Shahrekord Branch, Islamic Azad University, Shahrekord, Iran (Consent Ref Number IR.IAU.SHK.REC.1398.057). Verification of this research project and the licenses related to sampling process were approved by the Prof. Hassan Momtaz (Approval Ref Number MIC201946).

## AUTHOR CONTRIBUTIONS

Hassan Momtaz and Elahe Tajbakhsh carried out the molecular genetic studies, participated in the primers sequence alignment, and drafted the manuscript. Neda Noroozi and Elahe Tajbakhsh carried out the sampling and culture method. Hassan Momtaz and Elahe Tajbakhsh participated in the design of the study, performed the statistical analysis, and wrote the manuscript. All authors read and approved the final manuscript.

### PEER REVIEW

The peer review history for this article is available at https://publons.com/publon/10.1002/vms3.761.

## Data Availability

All data analyzed during this study are included in this published article.
